# Decomposition of Microbial Necromass Is Divergent at the Individual Taxonomic Level in Soil

**DOI:** 10.3389/fmicb.2021.679793

**Published:** 2021-07-02

**Authors:** Weiling Dong, Alin Song, Huaqun Yin, Xueduan Liu, Jianwei Li, Fenliang Fan

**Affiliations:** ^1^Key Laboratory of Biometallurgy of Ministry of Education, School of Minerals Processing and Bioengineering, Central South University, Changsha, China; ^2^Key Laboratory of Plant Nutrition and Fertilizer, Ministry of Agriculture and Rural Affairs, Institute of Agricultural Resources and Regional Planning, Chinese Academy of Agricultural Sciences, Beijing, China; ^3^Department of Agricultural and Environmental Sciences, Tennessee State University, Nashville, TN, United States

**Keywords:** decomposition, microbial necromass, whole community, soil, H_2_^18^O stable isotope probing

## Abstract

The turnover of microbial biomass plays an important part in providing a significant source of carbon (C) to soil organic C. However, whether the decomposition of microbial necromass (non-living microbial biomass) in the soil varies at the individual taxa level remains largely unknown. To fill up these gaps, we compared the necromass decomposition of bacterial and archaeal taxa by separating live microbial biomass with ^18^O-stable isotope probing from dead microbial biomass in soil. Our results showed that most of the microbial necromass at the operational taxonomic unit level (88.51%), which mainly belong to Acidobacteria, Actinobacteria, Gemmatimonadetes, and Proteobacteria, decomposed significantly after 30 days. In addition, there were great variations in necromass decomposition within each phylum, such as the decomposition of operational taxonomic units in Proteobacteria that ranged from 51% (*Beijerinckia*) to 92% (*Nitrosospira*). More importantly, the necromass decomposition was not related to the chemical composition of the cell wall but might positively correlate with the guanine–cytosine content of DNA and negatively correlated with genome size. This study provided a new insight that the decomposition of microbial necromass in soil was divergent at the individual taxonomic level and could not be fully explained by previously proposed mechanisms.

## Introduction

Carbon (C) transformation and sequestration in the terrestrial ecosystems have been studied broadly, as it is instructive for understanding the global C cycle and its role in climate change (Schmidt et al., 2011; Lehmann and Kleber, 2015). Soil organic matter is the largest C pool in the terrestrial ecosystems and acts as a critical component of global C cycling, which is relevant to climate change (Dixon et al., 1994; Schimel, 1995; Bellamy et al., 2005). It also has been studied that even small changes in global soil organic carbon (SOC) might lead to a significant influence on atmospheric carbon dioxide concentration (Davidson and Janssens, 2006; Chabbi et al., 2017). Although living microorganisms are only a small percentage of SOC, turnover of microbial biomass plays an important role in providing a significant source of C to SOC (Martin and Haider, 1979; Kindler et al., 2006; Simpson et al., 2007). In addition, the turnover of nonliving microbial biomass (necromass) is estimated to account for 80% of the soil organic matter pool and contributes a large flux of C into soil biogeochemical cycles (Liang and Balser, 2010; Fernandez et al., 2016; Kögel-Knabner, 2017). Therefore, it is significant to fill existing knowledge gaps of necromass decomposition for understanding C dynamics in the soil ecosystem.

Previous studies by adding selected groups of microbial isolates or their components suggested that the degradation of necromass was fast at the initial stage and depended on microbial identity (Nakas and Klein, 1979; Fernandez and Koide, 2012; Fernandez et al., 2019). DNA was used as a proxy of necromass constituents, and over 90% of the added DNA was lost during a 30-days incubation (Morrissey et al., 2015), but the addition of pure DNA to soils cannot give realistic results for native biomass. In addition, decomposition rates of microbial necromass from eight mycorrhizal fungal isolates (five isolates of Basidiomycetes and three isolates of *Cenococcum geophilum*) differed from each other (Fernandez and Koide, 2014; Fernandez et al., 2016). On the contrary, comparing the turnover rates of ^13^C-labeled necromass of some microbial isolates (i.e., Fungi, gram-negative bacteria, gram-positive bacteria, and Actinobacteria) suggested that the mineralization rates of necromass ^13^C did not depend on their identity (Throckmorton et al., 2012). These studies relied upon a limited number of microbial isolates. So far, no research has been conducted to assess the decomposition of necromass at the individual taxa level and compare them across whole microbial groups.

Both abiotic and biotic factors control the decomposition of necromass. Although it is well documented that necromass degradation is influenced by external factors such as drought, elevated carbon dioxide, and land-use change (Crowther et al., 2015; Ekblad et al., 2016; Ding et al., 2019), the intrinsic determinant of necromass degradation, e.g., the microbial characteristics of diverse microorganisms, is not well understood. The resistance of a cell wall and DNA to degradation were potentially controlling factors based on the consideration of the whole process of necromass decomposition. Microbial cell walls with a greater proportion of recalcitrant compounds, such as chitin in fungal cell walls and peptidoglycan mainly in gram-positive bacterial cells, are thought to be more resistant to decay their microbial necromass (Martin and Haider, 1979; Nakas and Klein, 1979; Kögel-Knabner, 2002). In addition, other components inside the cell, such as DNA, will decompose after cell lysis, and the degradation of DNA may also be affected by its physical property (such as genome size) and chemical composition. For instance, degradation of adenosine monophosphate by bacteria was much faster than cytidine monophosphate (Therkildsen et al., 1996), and microbial populations usually degrade DNA with low guanine and cytosine (GC) contents for acquiring N or P (Vuillemin et al., 2017). However, how the decomposition of necromass is affected by the factors mentioned earlier in soils at the individual taxa level remains unclear.

In the present study, we chose DNA as the proxy of necromass to investigate the degradation of bacterial and archaeal necromass at the individual taxa level. The primary objective of this study was to compare the necromass decomposition rates of microbial taxa. The potential microbial factors that might affect microbial necromass decomposition were also examined. To achieve these aims, the sterilized soil samples were inoculated with a small portion of unsterilized original soils for simulating an active soil condition and then incubated with H_2_^18^O. In particular, the H_2_^18^O stable isotope probing (SIP) approach was implemented to separate active microbes from the dead ones generated by gamma sterilization. We hypothesized that the decomposition rates of microbial necromass differed with large variations among different microbial taxa, which was governed by the chemical complexity of cell walls, genome size, and GC contents of DNA.

## Materials and Methods

### Soil Collection and Incubation

The surface (0–20 cm) Mollisol (United States Department of Agriculture Taxonomy) was collected from Hailun Agro-ecosystem Experimental Station of Chinese Academy of Sciences in Hailun City, Heilongjiang Province, China (47°26′N, 126°38′E) after maize harvest. The mean annual temperature is 1–2°C, but the mean temperature of the period of maize growing is more than 20°C, and the mean annual precipitation is 500–600 mm.

The air-dried soil sample was brought to 20% water (50% water holding capacity) and preincubated at 25°C for approximately 3 weeks to activate microorganisms. Then, the soil sample was passed through a 2-mm sieve, and subsamples (5.0 g) were put into 50-ml serum bottles before freeze-drying to remove H_2_^16^O in the LGJ-10C Lyophilizer for 24 h. The freeze-dried soil was sterilized with 40-kGy gamma radiation. Gamma sterilization was performed in the storage chamber using a ^60^Co gamma irradiator with rotation during irradiation to minimize variations in the absorbed dose, yielding a total absorbed dose of 40 kGy (0.8 kGy h^–1^ for 50 h). Each serum bottle with sterilized soil was inoculated with 0.05-g unsterilized soil (equal to 1% of the sterilized soil) to supply microbial decomposers for simulating microbial decomposition conditions in nature. Four soil subsamples stored at −80°C were used to determine the indigenous soil microbial diversity (i.e., day 0).

In general, there were two groups that each contain two treatments: (1) H_2_^16^O and ^16^O_2_ with inoculating unsterilized soil, H_2_^18^O and ^18^O_2_ with inoculating unsterilized soil; (2) H_2_^16^O and ^16^O_2_ without inoculating unsterilized soil, H_2_^18^O and ^18^O_2_ without inoculating unsterilized soil. The serum bottles covered with rubber lids and aluminum lids were flushed for 5 min with high purity nitrogen gas with a sterile needle and filter membrane to eliminate air in bottles. One-milliliter H_2_^18^O with 20% ^18^O_2_ (v/v) was added to the sterilized bottle using a sterilized syringe and a 0.2-μm filter. ^18^O_2_ was added to the bottle to eliminate the possible dilution of H_2_^18^O labeling by atmospheric oxygen gas. Soils were treated with H_2_^16^O, and 20% ^16^O_2_ (v/v) were used as controls. Twenty percent ^16^O_2_ or ^18^O_2_ (v/v) was supplied once a week. The second group of treatments incubating just for 30 days without microbial inoculation was included to check for the completion of sterilization. All treatments with four replicates were incubated at 25°C. Destructive sampling was conducted at 15 and 30 days, and soil samples were stored at −80°C.

### DNA Extraction, Density Gradient Centrifugation, and Gradient Fractionation

DNA was extracted using a FastDNA spin kit for soil (MP Biomedicals) following the manufacturer’s instructions. The extracted DNA was electrophoresed on 1.0% agarose gel, and the DNA concentration was measured by Nanodrop 2000 spectrophotometer (Thermo Scientific).

The DNA-SIP separation protocol was modified from previous studies (Neufeld et al., 2007). The extracted DNA (10 μg) was combined with gradient buffer (0.1-M *Tris*, 0.1-M potassium chloride, 1-mM ethylenediaminetetraacetic acid) and cesium chloride stock solution (1.89 g ml^–1^) to achieve a starting density of 1.725 g ml^–1^. The mixture was added to a 5.1-ml ultracentrifuge tube (Beckman, cat. no. 342412). Centrifugation was performed using an NVT-100 rotor in an ultracentrifuge (Beckman Coulter) at 177,000*g* for 30 h at 20°C. After centrifugation, an approximately 5-ml solution was separated into approximately 24 fractions and stored in 2-ml centrifuge tubes. The density of each fraction was measured with a digital refractometer (Reichert). Then, two volumes of polyethylene glycol solution (30% PEG 6000, 1.6-M sodium chloride) as precipitant were added to each 2-ml centrifuge tube to precipitate DNA from all fractions. The samples were then stored at room temperature overnight, and the DNA sample was separated from the solution by centrifugation at 13,000*g* for 30 min. The precipitate was washed with 300-μl 70% ethanol and suspended in 30 μl of sterile deionized water. The DNA concentration was measured by the Nanodrop 2000 spectrophotometer (Thermo Scientific).

The density-separated fractions of each sample were pooled according to the density distribution (light: 1.710–1.747 g ml^–1^; heavy: 1.753–1.790 g ml^–1^) to yield light and heavy fractions for further PCR and downstream analysis, respectively. The total light and heavy fractions after the H_2_^18^O treatments represented the DNA of microbial necromass and active microorganisms, respectively. The total light fraction in H_2_^16^O treatments represented the DNA of both dead and active microorganisms. Therefore, the decomposition of microbial necromass was done by analyzing the DNA of the total light fraction of the H_2_^18^O treatment.

### High-Throughput Sequencing and Real-Time Quantitative Polymerase Chain Reaction

Universal primers F515 and R806 were used for targeting the V3–V4 hypervariable region of 16S ribosomal RNA (rRNA) genes (Peiffer et al., 2013). Polymerase chain reactions (PCRs) were performed in triplicate 25-μl reactions containing 0.5-μl forward (F515) primer and reverse (R806) primer, 2-μl deoxynucleoside triphosphate, 2.5-μl PCR buffer, 0.25-μl rTaq polymerase, 18.25-μl sterile deionized water (H_2_O), and 1-μl DNA template. The thermal cycling conditions: included an initial denaturation step at 94°C for 5 min, followed by 30 cycles of denaturation at 94°C for 45 s, annealing at 55°C for 35 s, and extension at 72°C for 90 s. A final 10-min extension completed the PCR. Three PCR amplicons from each sample (technical replicates to reduce PCR bias) were pooled, and 2 μl of the pooled PCR products was used for the quantification of the DNA concentration with a Quant-it PicoGreen double-stranded DNA assay. Equal quantities of amplicons were pooled and purified with the TIANgel Midi Purification Kit (Tiangen Technologies). Sequencing was performed on a HiSeq2000 platform in paired-end mode (2 × 250 bp).

Raw sequence data were processed as reported by Dong et al. (2018). Sequences with quality scores greater than 20 and without mismatches in the barcode and primer regions were processed. The sequences were trimmed to 250 bp before clustering with UPARSE at a 97% similarity level (Edgar, 2013). Chimeras in the sequences were filtered with UCHIME (Edgar et al., 2011). The sequence analysis was performed using the USEARCH (version 8.1.1861) package containing UPARSE and UCHIME (Edgar, 2010). Representative sequences were classified on the RDP pipelin^1^. The sequences were deposited in the National Center for Biotechnology Information Sequence Read Archive under the accession number SRR9841183.

We performed quantitative PCR (qPCR) assays using the ABI prism 7000 machine and SYBR green to quantify microbial abundance obtained with primers that targeted regions of the 16S rRNA gene. Each 15-μl reaction mixture contained: 0.3-μl forward primer 357F and 0.3-μl reverse primer 518R, 7.5-μl SYBR Premix Ex Taq (TaKaRa), 0.3-μl Rox (TaKaRa), 4.6-μl sterile deionized H_2_O, and 2-μl DNA template. Standards were created by performing a 1:10 serial dilution to achieve a range from 10 to 10^8^ copies of the 16S rRNA gene. As described previously (Dong et al., 2018), the thermal cycle protocol included incubation at 95°C for 3 min, followed by 40 cycles of 95°C for 10 s, 52°C for 30 s, and 72°C for 45 s, with a final extension at 72°C for 10 min.

### Assessment of Sterilization Thoroughness by Plate Culture

Five grams of black soil samples (unsterilized or sterilized with 40-kGy gamma radiation after preincubation) was dissolved in 100-ml distilled deionized water and then homogenized by shaking for 20 min at 180 rpm. The suspension was allowed to stand at room temperature for 10 min to permit large insoluble particles to settle to the bottom of the conical flask. The supernatant was diluted (10^–5^ for control and 10^–1^ for sterilization) and used for plate coating using two bacterial growth media (beef extract peptone medium: 5 g L^–1^ beef extract, 10 g L^–1^ peptone, 5 g L^–1^ NaCl, 20 g L^–1^ agar; gause 1 medium: 1 g L^–1^ KNO_3_, 0.01 g L^–1^ FeSO_4_⋅7H_2_O, 0.5 g L^–1^ MgSO_4_⋅7H_2_O, 0.5 g L^–1^ K_2_HPO_4_, 20 g L^–1^ soluble starch, 0.5 g L^–1^ NaCl, 20 g L^–1^ agar). The plates were incubated at 30°C for 1 week.

### Assessing the Integrity of Microbial Cells by Scanning Electron Microscope

A scanning electron microscope (SEM) was used to assess the morphological stability of microbial cells in soil samples before and after sterilization. The six bacterial isolates (*Planococcus* sp., *Arthrobacter* sp., *Bacillus* sp., *Bacillus* sp., *Bacillus megaterium* sp., and *Burkholderia* sp.) were purified by streak culture of single colonies on plates with the unsterilized soil. Then, the bacteria were grown in a beef extract peptone medium without agar to the exponential phase and harvested in 2-ml centrifuge tubes by centrifuging at 8,000×*g* for 2 min. The six isolates in centrifuge tubes that sealed inside the cardboard box were used for gamma radiation. After that, six cultured isolates and their sterilized samples were prepared for the SEM analysis. The total 12 samples of bacterial pellets were fixed for at least 2 days in the glutaraldehyde fixative at room temperature. After fixation, the cells were washed with sterile deionized water three times. Subsequently, the cells were treated with an osmium-binding procedure for 2 h to increase their electrical conductivity in the SEM (Moore et al., 1978). Then, the cells were dehydrated in a graded ethanol series from 30 to 100%. The final dehydration was done in 100% ethanol overnight; then, the cells were critically point dried. The dried pellet was broken into small fragments with the tip of toothpicks and fixed onto an aluminum block using a conductive adhesive (Antony et al., 2011) and coated with a film of gold or gold–palladium using a sputter coater and then observed in an SEM.

### Data Analysis

The microbial necromass decomposition was calculated from the absolute abundance of the 16S rRNA gene of each bacterial and archaeal taxon at different incubation times, according to Fernandez and Koide (2012) and Carini et al. (2016). Only operational taxonomic units (OTUs) accounting for more than 0.05% of sequences at day 0 were selected for calculating the percentage of abundance loss. The decomposition of necromass (D), representing the percentage of abundance loss only from the sterilized soil during the incubation time, was calculated according to the following expression:

$$\begin{array}{c} D=\left(\frac{A_{T0}-A_{\text{Ti}}}{A_{T0}}\right)\times 100\%\\ \;=\left(\frac{F_{T0}\times P_{T0}-F_{\text{Ti}}\times P_{\text{Ti}}}{F_{T0}\times P_{T0}}\right)\times 100\% \end{array}$$

Where *A* represents the absolute abundance of 16S rRNA gene of each microbial taxa, *P* represents the proportion of 16S rRNA gene of each microbial taxa by sequencing, and *F* represents the number of total 16S rRNA gene copies by qPCR only from necromass in sterilized soil (DNA from the light fraction in H_2_^18^O treatment). T0 represents the time point at day 0, and Ti represents the time point at day 15 or 30.

Only OTUs with a *D* value > 0 were selected for further analysis. The high *D* value represents the decomposition with fast rates during the same period among different taxa (*n* = 4). The significant difference between the abundances of each OTU at day 15 or 30 and that at day 0 was assessed *via* t-test (*n* = 4; *P* < 0.05). The significant difference in the average decomposition between gram-positive (Actinobacteria and Firmicutes) and gram-negative (Acidobacteria, Gemmatimonadetes, and Proteobacteria) was assessed *via t*-test (*P* < 0.05).

The estimation of the genome size and the DNA GC content was processed as reported by Li et al. (2019). Briefly, we evaluated each unclustered, quality-filtered sequence for genome size and DNA GC content against a locally constructed complete genome database, and we compiled these into an estimate for each OTU. We used BLAST to identify which genome in the constructed database had the best match (defined by highest bit score) at the 16S rRNA locus to each environmental amplicon sequence. Genome size was estimated as the average size weighted by relative abundance in the database. The Pearson’s correlation of the GC contents and genome size with the decomposition (represented with *D* value) was performed by IBM SPSS Statistics (version 22.0.0.0).

## Results

### Technical Considerations

**FIGURE 1:**
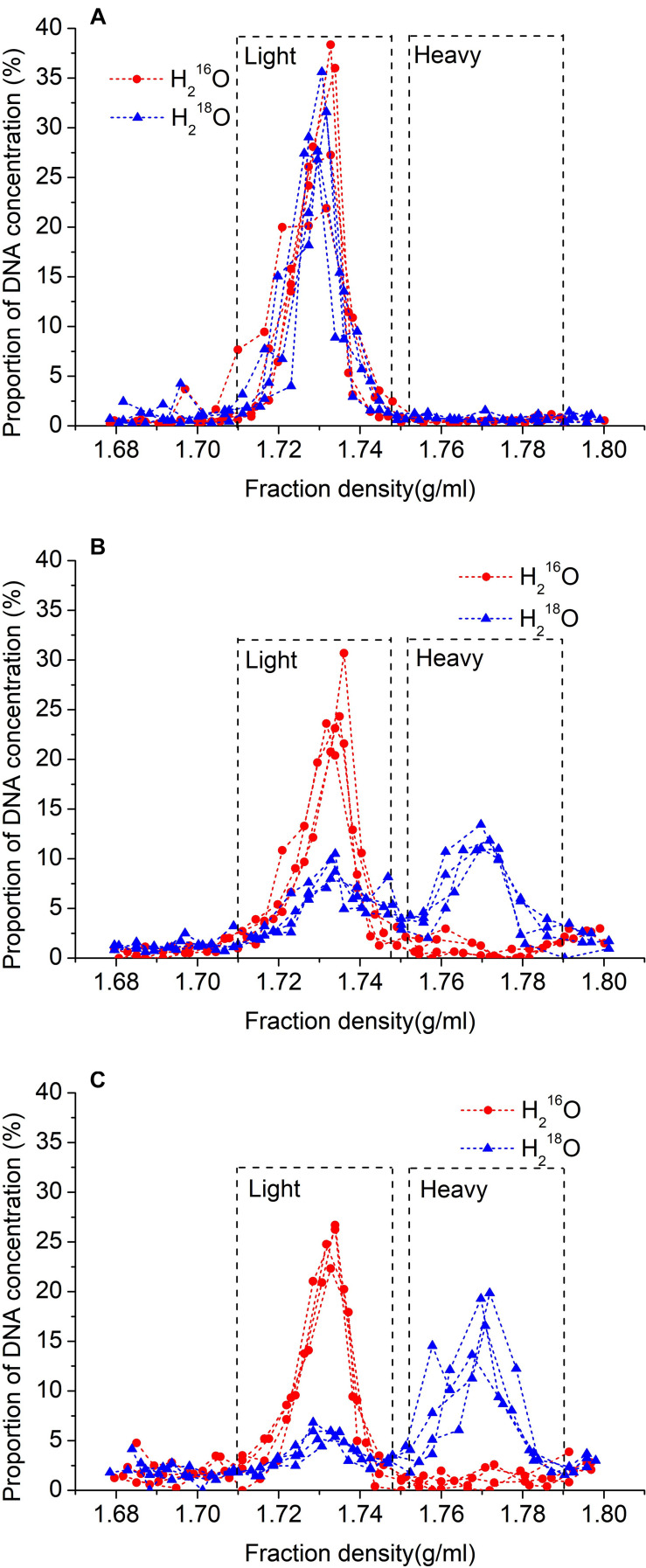
Relationship between density of fractions and proportion of total DNA recovered after 30 days without microbial inoculation **(A)**, and 15 days **(B)**, or 30 days **(C)** with inoculation after ultracentrifugation. ^18^O water replicates are represented by blue lines with triangles, and unlabeled replicates (control) are represented by red lines with circles.

The culture of the sterilized soil suspension on beef extract peptone or gause 1 medium plate at incubated 25°C for 1 week did not show any colony formation, suggesting that the sterilization was successful (Supplementary Figure 1). In addition, no heavy DNA was found in the sterilized soil without microbial inoculation after 30 days of incubation, confirming the success of the sterilization (Figure 1A).

After cesium chloride DNA gradient centrifugation, the labeled DNA (i.e., heavy fraction) ranged in density between 1.753 and 1.790 g ml^–1^, and the unlabeled DNA (i.e., light fraction) ranged between 1.710 and 1.747 g ml^–1^ (Figure 1). Also, the density of the peak DNA proportions in the heavy fraction was approximately 0.037 g ml^–1^ higher than that in the light fraction (Figure 1). In the samples treated with H_2_^18^O, 41.1% of the DNA was recovered in light fractions on day 15, whereas, on day 30, it was decreased to 27.0%.

**FIGURE 2:**
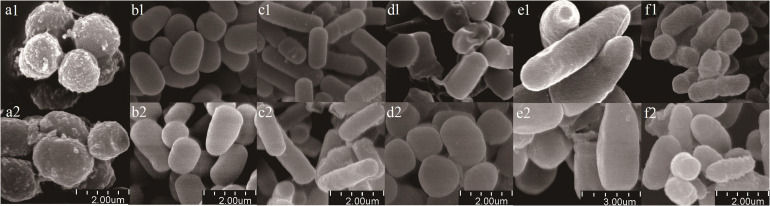
Scanning electron micrographs of bacterial isolates. (a) *Planococcus* sp.; (b) *Arthrobacter* sp.; (c) *Bacillus* sp.; (d) *Bacillus* sp.; (e) *Bacillus megaterium* sp.; (f) *Burkholderia* sp. Treatments were labeled by numbers (1 represents before sterilization, 2 represents after sterilization). Scale bars are shown at bottom of figure.

Scanning electron microscope micrographs showed that the six bacterial isolates (*Planococcus* sp., *Arthrobacter* sp., *Bacillus* sp., *Bacillus* sp., *Bacillus megaterium* sp., and *Burkholderia* sp.) existed as short rods, long rods, and spherical forms (Figure 2). Gamma sterilization did not induce morphological changes, such as surface damage such as cracks or wrinkles and obvious changes in sizes or shapes.

### Decomposition of Total Bacterial and Archaeal Necromass

**FIGURE 3:**
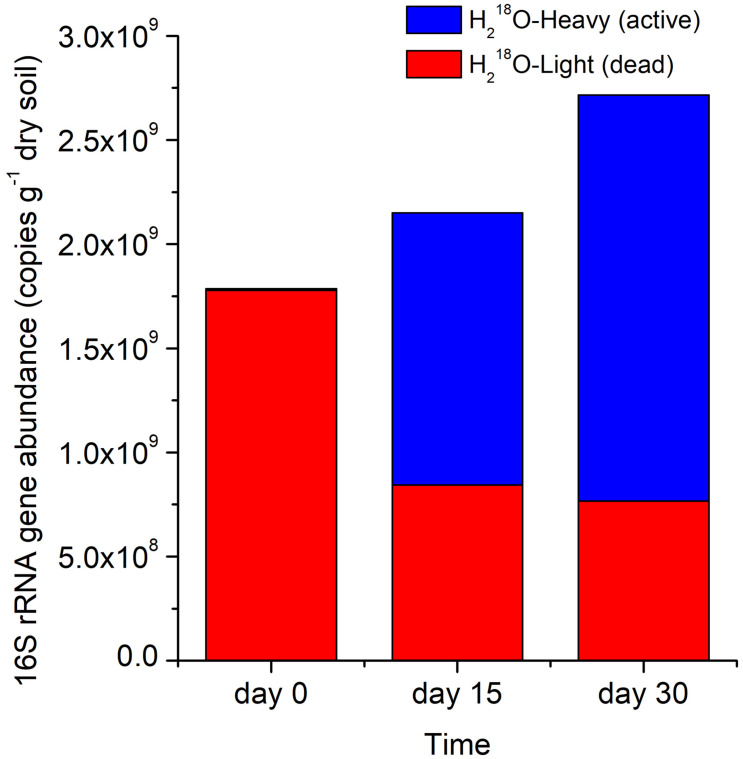
Stacked 16S rRNA gene abundance in H_2_^18^O heavy or light fraction at different times. Light and heavy fractions in H_2_^18^O treatments represented DNA of dead and active bacteria and archaea, respectively.

The copy number of the total 16S rRNA gene (active microbes and microbial necromass) in the H_2_^18^O treatment increased by 20.3% between day 0 and 15 and by 52.0% between day 0 and 30 (Figure 3). The 16S rRNA gene abundance of dead microbes representing microbial necromass (i.e., H_2_^18^O light fraction) declined rapidly by 52.5% after the first 15 days of the incubation but declined at a slower rate during the last 15 days (Figure 3).

### Microbial Necromass Decomposition at the Individual Taxonomic Level

**FIGURE 4:**
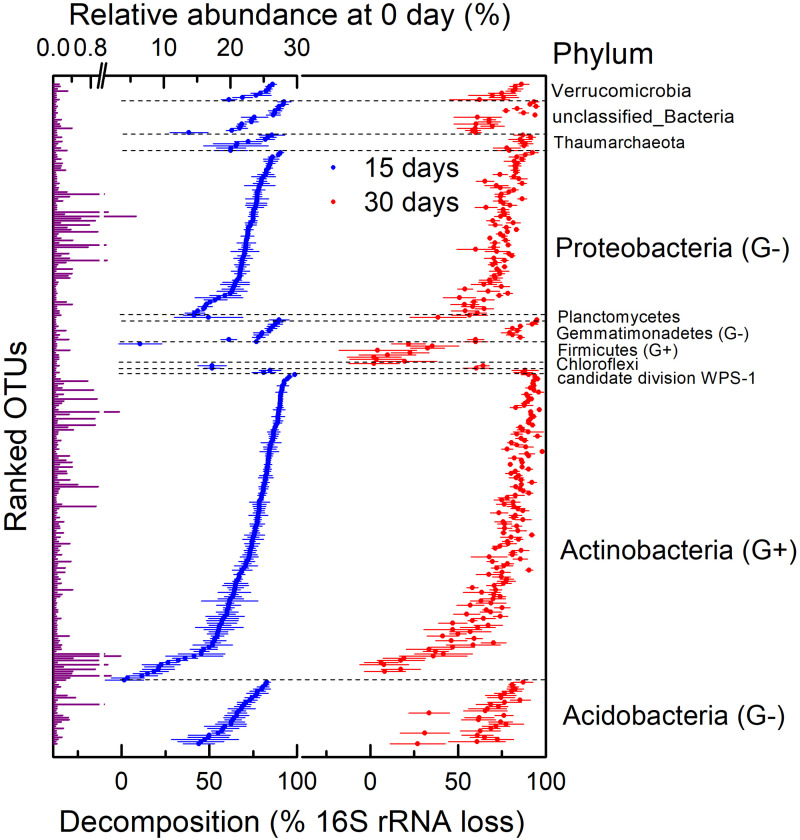
Relative abundance on 0 day and decomposition of 16S rRNA gene at operational taxonomic unit (OTU) level in H_2_^18^O light fraction (dead bacteria and archaea) at different times. This was assessed by calculating percentage of absolute abundance loss of 16S rRNA gene after 15 and 30 days. G+ in parentheses represents gram-positive bacteria, and G− in parentheses represents gram-negative bacteria. Points are average of four replicates, and bars show standard errors of means with four replicates.

We assessed the decomposition of necromass of microbial taxa by determining the absolute abundance of the 16S rRNA gene. First of all, bacterial and archaeal necromass accounting for 88.51% of total OTUs such as *Gp16*, *Blastococcus*, *unclassified_Sphingomonadaceae*, *Massilia*, and so on had a significantly lower abundance of 16S rRNA on day 30 than day 0 (*n* = 4; *P* < 0.05) due to a fast decomposition after 30 days of incubation (Figure 4). Other OTUs, mainly classified as *Arthrobacter*, *Streptomyces*, and *Nitrososphaera*, decomposed too slowly to be significant (*P* > 0.05). In addition, most of the OTUs in Firmicutes decomposed slowly during the 30 days of incubation. In general, necromass of OTUs mainly consisted of Acidobacteria, Actinobacteria, and Proteobacteria decomposed with different degrees after 30 days (Figure 4). The decomposition of OTUs in Actinobacteria ranged from 6% (OTU_6507 in *Streptomyces*) to 98% (OTU_354 in *Nocardioides*) on day 30.

### Factors Controlling Microbial Necromass Decomposition

There were large variations in the necromass decomposition within both gram-negative and gram-positive bacteria (Figure 4). The decomposition of gram-positive bacteria (Actinobacteria and Firmicutes) ranged from 2 to 98%, and decomposition of gram-negative bacteria (Acidobacteria, Gemmatimonadetes, and Proteobacteria) ranged from 27 to 95% on day 30. Some gram-positive taxa (e.g., OTU_138) decomposed faster than gram-negative taxa (e.g., OTU_1252), but some gram-negative taxa (e.g., OTU_161) decomposed faster than gram-positive taxa (e.g., OTU_105). In general, comparing the average decomposition of gram-positive and gram-negative bacteria showed no correlation between necromass decomposition and components of cell walls (Supplementary Figure 2; *P* > 0.05).

**FIGURE 5:**
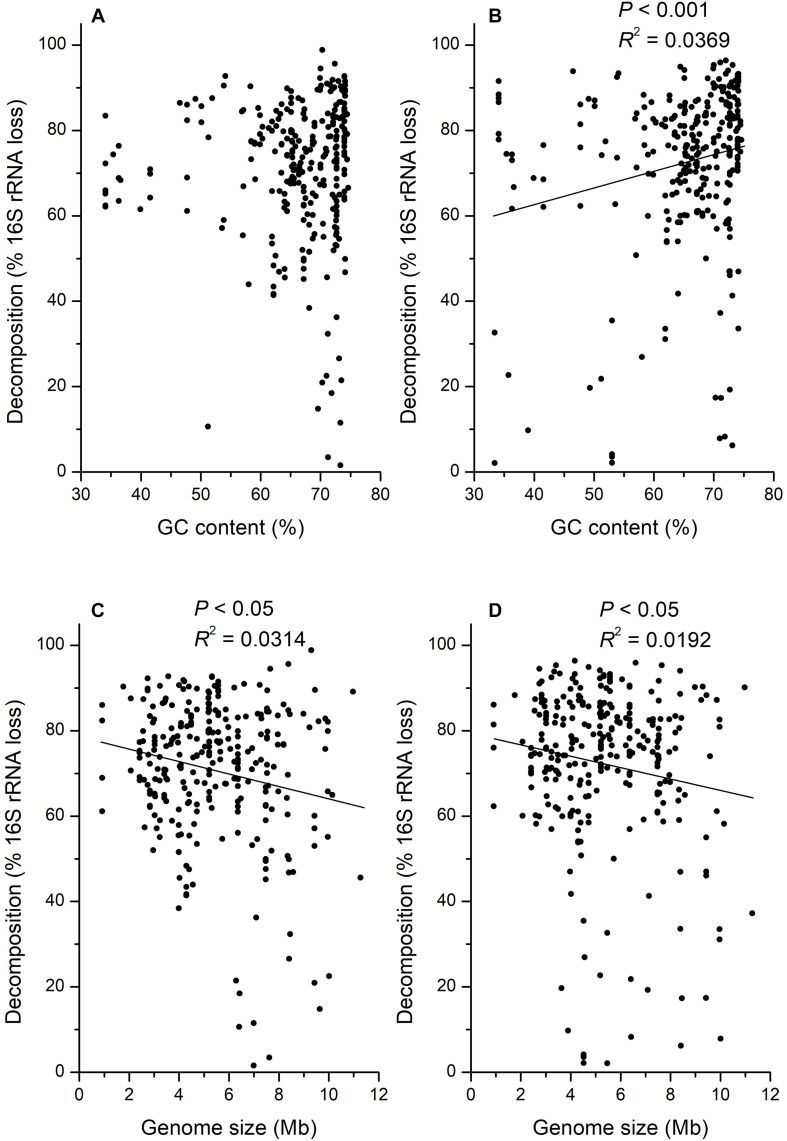
Correlation analysis for the decomposition of 16S rRNA gene in H_2_^18^O light fraction (dead bacteria and archaea) with GC content of DNA at 15 **(A)**, or 30 **(B)** days, and DNA genome size at 15 **(C)**, or 30 **(D)** days.

There was also a large variation in the relationship between decomposition and GC contents. Some taxa (e.g., OTU_99: 65.1% GC) with high GC contents decomposed faster than taxa (e.g., OTU_81: 47.7% GC) with low GC contents, but some taxa (e.g., OTU_37: 63.0% GC) with low GC contents decomposed faster than taxa (e.g., OTU_83: 72.6%) with high GC contents. Generally, the decomposition on day 15 was not significantly correlated with GC contents (Figure 5A; *P* > 0.05) but positively correlated with GC contents on day 30 (Figure 5B; *P* < 0.001, *R*^2^ = 0.0369). However, the decomposition on both day 15 (*R*^2^ = 0.0314) and 30 (*R*^2^ = 0.0192) was significantly negatively correlated with microbial genome sizes (Figure 5; *P* < 0.05), whereas these correlations may be weak.

## Discussion

Here, we aimed to study decomposition and its potential factors of individual taxa of the whole bacterial and archaeal community of microbial necromass retaining interactions with its soil environments. Overall, the results of the whole experiment support part of our hypothesis that decomposition rates for microbial necromass would differ among microbial taxa. However, it is surprising that the results of the present study did not support the hypothesis completely that microbial necromass decomposition was correlated with the chemical complexity of cell walls, genome size, and GC contents of DNA.

We calculated the percentage of absolute abundance loss of 16S rRNA gene, which accounted for 52.5 and 57.0% on day 15 and 30, respectively (Figure 3), suggesting that the necromass of the whole bacterial and archaeal community decomposed rapidly and mainly in the first 15 days, which was consistent to researches that the degradation of necromass was fast at the initial stage (Fernandez and Koide, 2014; Morrissey et al., 2015). The gradually slow decomposition of the microbial necromass with incubation time may result from that DNA released from cells was adsorbed by surface-reactive soil particles, thus being protected against degradation by nucleases in soil (Cai et al., 2006; Pietramellara et al., 2009; Schmidt et al., 2011; Saeki et al., 2012).

However, there was a large variation in necromass decomposition within the phyla (mainly Acidobacteria, Actinobacteria, and Proteobacteria), and the decomposition of OTUs in Proteobacteria ranged from 51% (OTU_139 in *Beijerinckia*) to 92% (OTU_161 in *Nitrosospira*), indicating that the decomposition of microbial necromass is related to the microbial taxa (Figure 4). Most of the microbial necromass at OTU level (88.51%, mainly belong to genera *Gp16* of Acidobacteria, *Blastococcus*, *unclassified_Sphingomonadaceae*, *Massilia*, and so on) decomposed significantly after 30 days, whereas some OTUs (mainly classified as *Arthrobacter*, *Streptomyces*, *Nitrososphaera*, and so on) were relatively recalcitrant. Therefore, the decomposition of microbial necromass was taxa-specific, contradicting reports that there is no correlation between the degradation of microbial necromass and the source of microbial C in soil (Throckmorton et al., 2012) but confirming what found for the decomposition of necromass of several mycorrhizal species, which depended on the melanin concentration of the ectomycorrhizal necromass (Fernandez and Koide, 2014; Fernandez et al., 2016). Such discrepancies among different studies suggest that the results of microbial necromass decomposition were affected by the selected microbial groups that were isolated from soil to generate necromass. Although it was difficult to isolate lots of microorganisms in soil because most of them were not culturable in the laboratory (Rappe and Giovannoni, 2003; Lloyd et al., 2018), it was necessary to study the decomposition of necromass as many kinds of microbes as possible. Consequently, it is important to underline that our study presented the whole pattern of the necromass decomposition of the total bacterial and archaeal community in soil by quantifying it in the individual taxonomic level.

We also studied the potential role of the chemical composition of cell walls among microbial taxa on the decomposition of the microbial necromass. Because DNA needs to be released to the extracellular environment through the broken cell wall for further decomposition, we infer that a resistant cell wall would prevent DNA from entering the soil environment and reduce its decomposition rate. It is usually believed that the turnover of microbial cell walls containing more complex and recalcitrant polymerize components is relatively slow (Kögel-Knabner, 2002; Strickland and Rousk, 2010). In our study, the decomposition at OTU level of both gram-positive and gram-negative bacteria was variable, but there was no significant difference in the average decomposition between these two bacterial groups at 15 or 30 days (Supplementary Figure 2; *P* > 0.05). This contradicts some reports that gram-positive bacteria may be less easily decomposed than gram-negative bacteria because they have a higher peptidoglycan content in their cell walls (Kögel-Knabner, 2002; Strickland and Rousk, 2010) but confirms what was reported by Throckmorton et al. (2012) and Joergensen (2018) that microbial necromass quality did not affect the turnover rate of microbial necromass. It is possible that, unlike plant litter that consists of complex components, including decompose-resistant lignin and aromatic (Aerts, 1997; Derenne and Largeau, 2001), the relatively simple chemical properties of microbial necromass were not enough to make significant effects on its decomposition. Besides, the range of the stoichiometric ratio of microbial taxa such as C:N is much narrower than that of plant litters might lead to its unavailability as a controlling factor for decomposition in our study (Enriquez et al., 1993). At last, as we observed little decomposition of some OTUs in Actinobacteria and Firmicutes, the variation in the cell envelope such as spore formation might also protect necromass from decomposition.

Besides, the physical and chemical properties of microbial DNA may also influence its decomposition. Because microbial degradation of adenosine monophosphate is faster than cytidine monophosphate (Therkildsen et al., 1996), we expected microbes with lower GC contents in DNA sequences would decompose faster than those with higher GC contents because DNA can be used as a nutrient source by soil microorganism (Dell’Anno et al., 2002; Pietramellara et al., 2009; Vuillemin et al., 2017). However, correlation analysis showed that only the decomposition on day 30 was significantly positively correlated with the GC contents (Figure 5B; *P* < 0.001), which was opposite to our assumption based on previous studies. It was necessary to monitor the correlation between the decomposition and GC contents for a longer incubation time to confirm it, as the significance was not observed on day 15. Consequently, the GC contents might be a related but not a critical determinant of microbial necromass decomposition. For the physical property of DNA, it is interesting that the microbial genome size was negatively correlated with the necromass decomposition in our study, as there was no research associated with it before (Figure 5; *P* < 0.05). One possible reason leading to this phenomenon is that microbial necromass with high genome size has a smaller surface-to-volume ratio, which may lead to the reduction of decomposition efficiency. As it has been reported that the microbes with low genome size usually have small cell size (Dill et al., 2011) but larger surface-to-volume ratios for uptake of nutrients (Dufresne et al., 2005; Sabath et al., 2013). In addition, microbial necromass with high genome size needs more time to decompose until that could not be detected than low genome size under the same decomposition efficiency.

We chose gamma radiation sterilization because autoclaving sterilization is more destructive of soil physical and chemical properties, including polysaccharide structures holding the microaggregates together (McNamara et al., 2003; Berns et al., 2008). The result of H_2_^18^O incubation in ^18^O_2_-substituted air without microbes revealed that there was no DNA in the heavy fraction in both control and isotope treatments (Figure 1C), which, together with no colony on the plate with sterilized soil, indicated that our sterilization was complete (Supplementary Figure 1). Also, the density of the peak DNA proportions in the heavy fraction was approximately 0.037 g ml^–1^ higher than that in the light fraction, suggesting that almost all active microbes using H_2_^18^O were separated from the light fraction (Figure 1). The result of SEM showed that the effect of 40-kGy gamma radiation on cellular morphology did not influence the cell integrity of microbes (Figure 2). In addition, the density change between light and heavy fractions was sufficient to separate unlabeled and labeled DNA (Figure 1). Besides these accurate methods performed in the experiment, we removed OTUs that have very low relative abundance (<0.05%) and abnormally calculated decomposition (<0%) before formal analysis to avoid errors (Bokulich et al., 2013; Carini et al., 2016; Schöler et al., 2017).

However, there are still deficiencies in our study. The inoculation with unsterilized soil (1% of the sterilized soil) would bring some dead and dormant cells simultaneously, which may lead to the most 1% error in calculating the decomposition of necromass generated by sterilization. Besides, cell death can occur by physical damage of the cell and spontaneous or pathogen-induced cell lysis and necrosis (Levy-Booth et al., 2007; Pietramellara et al., 2009). Necromass caused by gamma sterilization might be different from natural cell lysis caused by starvation or by phages in natural soil conditions, although gamma sterilization is probably one of the best artificial ways currently. Therefore, it will be much interesting to compare decompositions of necromass generated in different ways. In addition, it is probably that cell cycles of some members like Actinobacteria and Firmicutes by spore formation would prolong the whole-cell cycles, including active, dormant, and dead period of cells (Mayfield et al., 1972; Barka et al., 2015), which would decrease turnover of their biomass. Consequently, it is important to study the effect of spore formation on the decomposition of necromass.

## Conclusion

Although microbial biomass turnover has been studied widely for its importance in representing soil organic matter turnover, knowledge gaps existed on the decomposition of microbial necromass at individual taxa. Our study provided a direct assessment of the necromass decomposition rates of the whole bacterial and archaeal community and the potential factors affecting their decomposition in black soil. Generally, our results support the hypothesis that the decomposition rates of microbial necromass depend on the taxa. The necromass of some taxa, such as *Blastococcus*, *Gemmatimonas*, and *Massilia*, was decomposed fast during incubation, but that of other taxa, such as *Singulisphaera*, *Streptomyces*, and *Nitrososphaera*, was relatively recalcitrant to be decomposed. We infer that microbial biodiversity is likely to influence the persistence of microbial necromass in soil, and communities dominated by taxa with recalcitrant necromass might have more accumulated soil C from necromass. In addition, necromass decomposition was not related to the chemical composition of the cell wall but might be affected by the genome size and GC content of DNA. Finally, further research should also concern the necromass decomposition of the whole fungal community *in situ* to enrich the knowledge of microbial necromass decomposition.

## Data Availability Statement

The sequences in this study were deposited in the National Center for Biotechnology Information (NCBI) Sequence Read Archive (SRA) under the accession number SRR9841183.

## Author Contributions

WD: investigation, formal analysis, and writing–original draft. AS, HY, JL, and XL: writing–review and editing. FF: conceptualization, supervision, and writing–review and editing. All authors contributed to the article and approved the submitted version.

## Conflict of Interest

The authors declare that the research was conducted in the absence of any commercial or financial relationships that could be construed as a potential conflict of interest.

## Footnotes

1 http://rdp.cme.msu.edu/
