# Supplementary material for: Decomposition of Microbial Necromass Is Divergent at the Individual Taxonomic Level in Soil
Source: Front Microbiol. 2021 Jul 2;12:679793. doi: 10.3389/fmicb.2021.679793 (PMC8283313; doi:10.3389/fmicb.2021.679793)
Supplement: Supplementary file 1 [file Data_Sheet_1.docx]

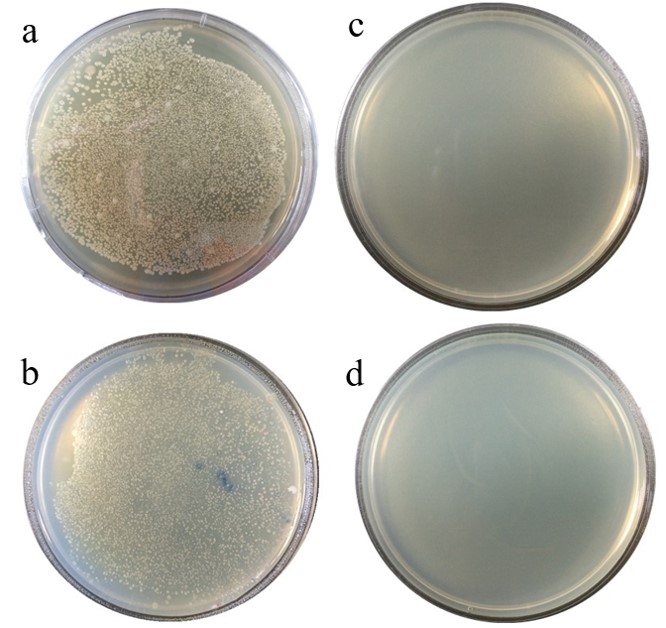


**Figure S1.** Colonies of bacteria grown on the plates. Soil suspension was plated on beef extract peptone (**a**) or gause 1 (**b**) medium from 0 kGy gamma radiation sterilization soil, and was plated on beef extract peptone (**c**) or gause 1 (**d**) medium from 40 kGy gamma radiation sterilization soil.


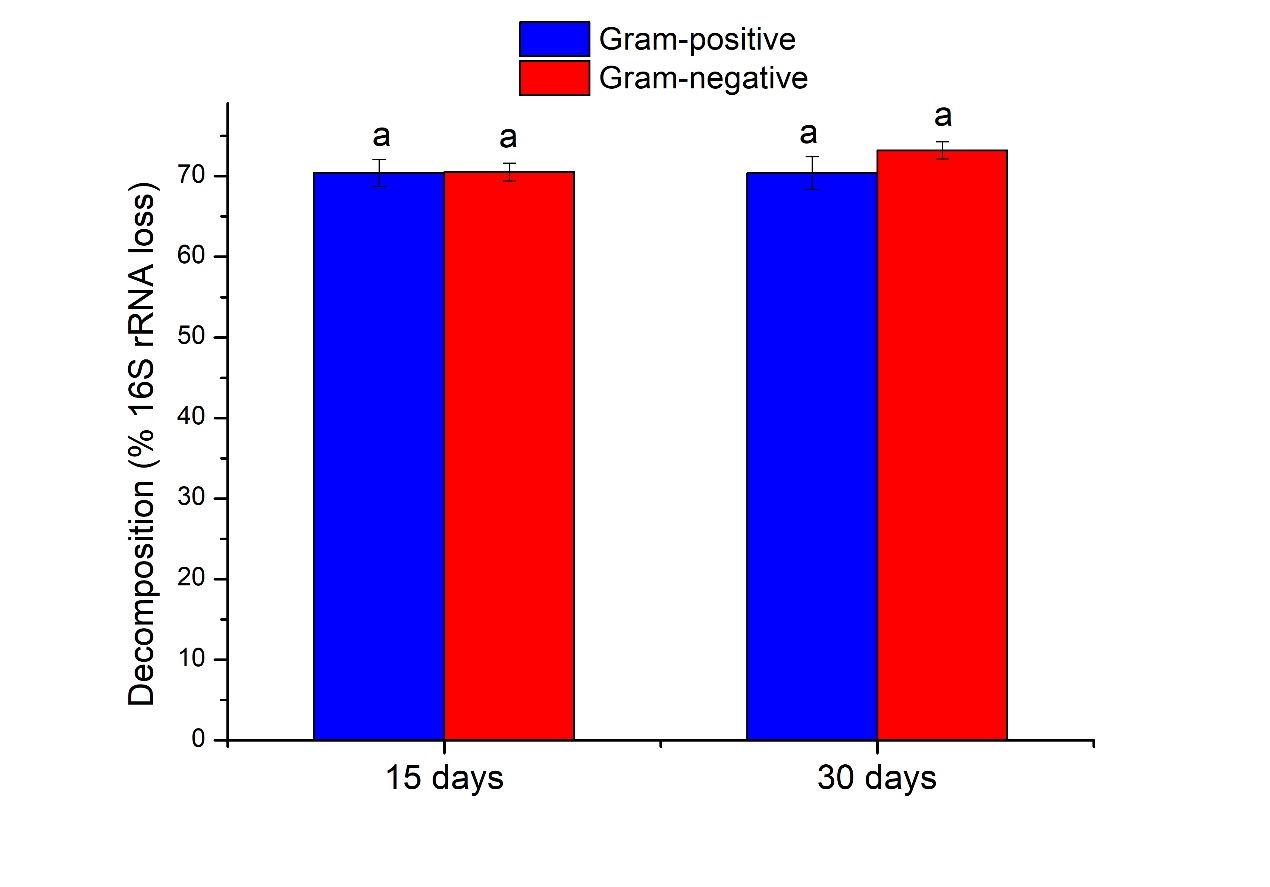


**Figure S2**. Average decomposition of Gram-positive and Gram-negative bacteria in the H_2_^18^O light fraction (dead bacteria) at different times. Different letters indicate significant differences between Gram-positive and Gram-negative bacteria at the same timepoint, and bars show standard errors of the means with 4 replicates.
